# Combined Application of Malic Acid and Lycopene Maintains Content of Phenols, Antioxidant Activity, and Membrane Integrity to Delay the Pericarp Browning of Litchi Fruit During Storage

**DOI:** 10.3389/fnut.2022.849385

**Published:** 2022-03-17

**Authors:** Hua Huang, Ling Wang, Fangcheng Bi, Xu Xiang

**Affiliations:** ^1^Institute of Fruit Tree Research, Guangdong Academy of Agricultural Sciences; Key Laboratory of South Subtropical Fruit Biology and Genetic Resource Utilization, Ministry of Agriculture and Rural Affairs, Guangdong Provincial Key Laboratory of Tropical and Subtropical Fruit Tree Research, Guangzhou, China; ^2^Sericultural & Agri-Food Research Institute Guangdong Academy of Agricultural Sciences, Key Laboratory of Functional Foods, Ministry of Agriculture and Rural Affairs, Key Laboratory of Agricultural Products Processing, Guangzhou, China

**Keywords:** litchi fruit, pericarp browning, phenols, antioxidant activity, membrane integrity

## Abstract

Litchi fruit contains abundant polyphenols and is susceptible to browning after harvest. Herein the combined treatments of malic acid (MA) and lycopene (LYC) to delay the development of browning in litchi fruit stored at room temperature (25°C) and low temperature (4°C) was investigated. The results showed that the pericarp browning could be alleviated, and the increase of malondialdehyde (MDA) content and relative leakage rate was retarded by the combined MA and LYC during storage. As compared to control, the content of pericarp anthocyanins, flavonoids, and the total phenols maintained higher levels; and the decrease of antioxidant activity of 2,2-diphenyl-1-picrylhydrazyl (DPPH) radical scavenging capacity and reducing power were slowed down in treated fruit. The enzyme activity of polyphenol oxidase (PPO) and peroxidase (POD) related to oxidation of polyphenols were depressed by the combined treatments. Furthermore, correlation analysis revealed that the content of phenols in the pericarp negatively affected the changes in the browning index, and was positively related to the DPPH radical scavenging capacity. Taken together, the combined treatments of MA and LYC exhibited potential effects in delaying the pericarp browning of litchi fruit by maintaining the content of polyphenols, antioxidant activity, and membrane integrity.

## Introduction

Litchi (*Litchi chinensis* Sonn.) is a typical subtropical to tropical fruit and is popular o be consumed for its nutritional and tender aril flesh (1). However, litchi is sensitive to environmental factors and develops pericarp browning rapidly once the fruit is detached from the tree. The browning in litchi fruit is largely impacted by the oxidation of its abundant polyphenols (2), energy dissipation (3), and changes of water status (4). The oxidation of polyphenols is well-known for the enzymatic browning in litchi fruit pericarp. Polyphenol oxidase (PPO) (EC 1.10.3.1) and peroxidase (POD) (EC 1.11.1.7) have been widely reported to be the main way for oxidation browning. PPO involves the co-oxidation of phenols and anthocyanins to *o*-quinones, while POD functions with the presence of H_2_O_2_ reaction to form browned polymers (2, 5). Therefore, postharvest inhibition of rapid oxidation of polyphenols is important to delay the senescence of litchi fruit.

Numerous methods and technologies have been explored to inhibit the rapid senescence process in litchi after harvest. These methods and technologies include physical treatment of sulfur dioxide (6) or packaging (7); application of antioxidants, i.e., apple polyphenols and α-lipoic acid (8, 9); and film covering to maintain the water status by chitosan (10). However, the application of appropriate treatments together with low-temperature (LT) storage is necessary to be further explored for the storage of litchi fruit.

The application of organic acid on postharvest crops in extending shelf-life has been widely mentioned. For instance, the treatments of oxalic acid retard the rapid pericarp browning in litchi (11), and salicylic acid alleviates the browning in banana fruit (12). Citric acid is usually used as an enhancer together with other treatments to maintain the postharvest quality in crops (13). These reports indicate that organic acid treatments could enhance the antioxidant activity to slow down the senescence of the horticulture crops. Malic acid (MA) is an organic acid produced in almost all living cells and is widely used as a food additive. It also has been reported that MA could be a single treatment to alleviate chilling injury in banana fruit (14), or combined with citric acid to extend the vase life of cut lily flower (15), or together with oxalic acid to preserve the quality of pomegranate fruit after harvest (16). It indicates that MA might be also one of the organic acid candidates for the application on postharvest crops. Lycopene (LYC) is a natural antioxidant made in many living organisms and useful for food coloring and health constituents. As one of the main nutritional components in horticulture crops, i.e., tomatoes and carrots, the changes in LYC have been used to monitor the ripening process (17). In addition, LYC as a natural antioxidant was reported to be efficient in extending the shelf-life of linseed oil and cow milk fat (18, 19). However, the potential effect of LYC on the postharvest changes of litchi fruit has not yet been explored.

This study was set to explore the application of MA and LYC in affecting the browning in litchi fruit under room and LT storage. Consider the previous application of MA was used as to enhance element, in this study, litchi fruit was treated by MA followed by LYC in series. The effect of the combined treatments on development of pericarp browning, the content of phenols, anthocyanins, and flavonoids; 2,2-diphenyl-1-picrylhydrazyl (DPPH) radical scavenging capacity and reducing power; and the membrane integrity were thoroughly conducted. Results obtained from this study will help to further explore the potential effect of MA combination natural antioxidants to delay the development of browning in litchi.

## Materials and Methods

### Fruit Materials

Litchi fruit (*Litchi chinensis* Sonn. cv. “Huaizhi,” HZ) at the mature stage were harvested from Guangzhou Litchi Resource Orchard, Guangzhou, P. R. China (23°30′N, 113°30′E). Litchi fruit was picked up at the pink-red stage (about 80 days after anthesis). Fresh fruit was packed with 0.03 mm bags and immediately transported to the laboratory within 2 h. Fruit with uniformity of shape, color, and without cracks was carefully selected. The selected fruit was then immersed in 0.1% prochloraz for 3 min to protect it from pathogen infection. Preliminary treatments of MA with 10, 20, 40, and 80 mM, and LYC with 0.1, 0.2, 0.4, and 0.8 mM were performed. Fruit treated with MA at 40 and 80 mM, with LYC at 0.2 and 0.4 mM showed potential in delaying the browning of litchi fruit. MA and LYC were both obtained from Beijing Solarbio Science & Technology Co., Ltd. Therefore, in this study, fruit were treated with 40 mM MA (dissolved in water, W/V) for 5 min followed by dipping in 0.4 mM LYC (first dissolved in 10 ml pure ethanol with tween 20) for 5 min. Fruit dipped in water only for 10 min was set as control. After air-dried (1–2 h) at room temperature (RT), the fruit was randomly packed in 0.03 mm polyethylene bags with 16 fruits per bag, three parallel bags were packed as the replications at each treatment for each time point. The fruit was stored at room ambient (around 25°C with 75–90% humidity) and LT (4°C). Samples were assayed at 0, 2, 4, and 6 days under RT and 10, 20, and 30 days under LT, respectively. After evaluating the pericarp browning index and the relative leakage rate, pericarp tissues were frozen in liquid nitrogen, crushed in small pieces, and stored at −80°C for further experiments.

### Determination of the Browning Index

Changes in pericarp browning were evaluated visually by calculating the overall browned surface area (20). In total 48 fruits in three bags as replicates at each treatment and each point were analyzed. The visual appearance scale for the pericarp browning was set as: (1) no browning (fresh fruit); (2) slight browning lower than one-fourth surface; (3) one-fourth to one-half browning; (4) one-half to three-fourths browning; and (5) >three-fourths browning. Based on the recorded browning scales, browning index (BI, indicating the overall browning changes) could be calculated as:


$$\begin{array}{l} \text{Browing index}=\\ \frac{\sum \text{browning scale }\times \text{ number of fruit in each scale}}{\text{total number of fruit}} \end{array}$$

### Assay of MDA Content and Relative Leakage Rate

The membrane integrity was indicated by the content of malondialdehyde (MDA) and relative leakage rate (9). Briefly, frozen pericarp tissues were ground using an FW-100 grinder (Yongguangming Medical Equipment Co., Ltd., Beijing, China), and 2.0 g powder was homogenized with 10 ml of trichloroacetic acid (TCA) (10%, W/V). The mixtures were centrifuged at 13,000 × *g* for 15 min at 4°C and collected the supernatant. Then, 1 ml of supernatant was added in 3 ml 0.5% thiobarbituric acid (TBA). The mixtures were boiled for 20 min and cooled down on an ice bath and centrifuged with 13,000 × *g* for 10 min at 4°C. Absorbance values of supernatant were measured under 450, 533, and 600 nm, respectively, by a spectrophotometer (UVmini-1240, Shimadzu Corporation, Japan). MDA content was calculated according to [6.45 × (A_532_–A_600_)]–[0.56 × A_450_]. Results were expressed as μmol g^−1^ on basis of fresh weight (FW).

The electrolyte leakage rate was indicated by the electric conductivity and determined according to the previously reported method (21). Pericarp discs were obtained using a puncher with a diameter of 1.2 cm. Discs were washed with distilled water three times, and 15 discs were prepared for each replicate, three replicates for each sample point were prepared. Discs were then immersed in 20 ml distilled water and incubated for 2 h at 25°C. Then, the relative leakage rate (R_1_) of discs was measured by a conductivity meter (Model DDS-llA, Shanghai Scientific Instruments). After that, disks in distilled water were boiled for 15 min and cooled on an ice bath. Then, the relative leakage rate (R_2_) of boiled samples was measured again. The electrolyte leakage rate was calculated as the ratio of R_1_ vs. R_2_.

### Determination of Content of Total Phenols, Flavonoids, and Anthocyanins

The overall content of phenols in the litchi pericarp tissues was measured according to the previously reported method (22). Frozen pericarp tissues ground in powder of 2.0 g was randomly taken and dissolved in 20 ml methanol. Ultrasonic-assisted extraction was set for 30 min under 50°C. Three replicates were set at each time point in each treatment. The mixtures were centrifuged at 13,000 × *g* for 30 min and collect the supernatant. To measure the total phenols, 0.5 ml supernatant was added in 5 ml distilled water containing 0.5 ml Folin–Ciocalteu reagent. After incubated for 8 min at RT, 1.5 ml sodium carbonate solution (20%, W/V) was added, and the mixtures were incubated for 30 min under RT and dark. Absorbance at 760 nm was measured (UVmini-1240, Shimadzu Corporation, Japan). Gallic acid at different concentration ranges (0–100 mg l^−1^) was used as the standard for the calibration curve. Total phenols were calculated and expressed as mg of the gallic acid equivalents per gram of samples (mg g^−1^) on basis of FW.

The changes in flavonoids content were determined according to the previous method with minor modifications (23). Frozen tissues ground in powder of 2.0 g were mixed with 20 ml ethanol for 1 h under RT and then filtered to collect the supernatant. To detect the overall content of flavonoids, 1 ml extract solution was diluted with 3.5 ml distilled water, and then 0.5 ml 5% NaNO_2_ was added. After 6 min, 0.5 ml 10% AlCl_3_ was added and incubated udder RT for 6 min. Then, 4 ml 4% NaOH was added and incubated for 15 min under RT. Absorbance at 510 nm was measured (UVmini-1240, Shimadzu Corporation, Japan). Different concentrations of catechin were used as standard. The flavonoids content was expressed as mg catechin equivalents per gram of samples (mg g^−1^) on basis of fresh weight.

The content of anthocyanins was determined using the method of Zhang et al. (5) with minor modifications. Frozen tissues ground in powder of 2.0 g were mixed with 20 ml 0.1 M HCl shaking for 24 h under RT. After centrifuged at 13,000 × g for 10 min at 4°C, 0.5 ml supernatant was added in 2.5 ml 0. 4 M KCl (pH 1.0), and 2.5 ml 0.4 M citric acid-Na_2_HPO_4_ buffer (pH 5.0), respectively. Absorbance at 510 nm for the mixtures at different pH was measured (UVmini-1240, Shimadzu Corp., Japan). Anthocyanin content was calculated as cyanidin-3-glucoside calibration, expressed as mg g^−1^ on basis of fresh weight.

### Determination of Enzyme Activity of PPO and POD

To analyze the activity of enzymes related to oxidation of phenols, frozen tissues ground in powder of 2.0 g were mixed with 20 ml 0.2 M phosphate buffer solution (pH 7.0) containing 2% polyvinylpolypyrrolidone (PVPP). After centrifuging for 10 min at 13,000 × g and 4°C, supernatants were collected and used to determine the enzyme activities (3).

Polyphenol oxidase activity was measured by the oxidation of catechol as a substance. Briefly, the reaction was initiated by adding 0.1 ml of supernatant in 2.9 ml reaction buffer (0.05 M sodium phosphate, pH 7.0) containing 10 mM catechol. Changes of absorbance at 398 nm were recorded every 30 s for 4 min (UVmini-1240, Shimadzu Corporation, Japan). One unit of PPO activity was defined as 0.001 changes in absorbance per minute. PPO activity was expressed as U g^−1^ on basis of fresh weight.

Peroxidase activity was measured according to the oxidation of guaiacol by the presence of H_2_O_2_. The reaction buffer containing 0.1 ml 4.0% guaiacol, 0.1 ml 0.46% H_2_O_2_ and 2.75 ml 0.05 M sodium phosphate (pH 7.0) was prepared. The oxidation reaction was initiated by adding 0.05 ml above rude enzyme solution. Changes of absorbance at 470 nm were recorded every 30 s for 4 min (UVmini-1240, Shimadzu Corp., Japan). One unit of POD activity was defined as 0.01 changes in absorbance per minute. POD activity was expressed as U g^−1^ on basis of fresh weight.

### Assay of DPPH Scavenging Activity and Reducing Power

The changes of non-enzymatic antioxidant activity of fruit pericarp were indicated by scavenging activity of DPPH and reducing power (24). To prepare the reaction solution, frozen tissues ground in powder of 2.0 g were added to 20 ml methanol with ultrasonic-assisted extraction for 30 min at 50°C. Three replicates were set at each time point for each treatment. The mixtures were then centrifuged at 13,000 × *g* for 30 min and collected supernatants for further antioxidant activity analysis.

To determine the scavenging activity of DPPH radicals, 0.1 ml of methanol-extracts was mixed with 2.9 ml of 0.1 mM DPPH (dissolved in methanol). The reaction mixtures were incubated at 25°C under dark for 20 min. Absorbance at 517 nm was measured (UVmini-1240, Shimadzu Corp., Japan). Samples with all reaction mixtures without the above extracts were used as control, and without DPPH were used as blank. The scavenging activity of DPPH radicals was expressed as a percentage (%): (1–absorbance of sample/absorbance of control) ×100.

Reducing power was assayed by adding 0.1 ml of the above methanol extract to 2.5 ml of 0.1 M phosphate buffer (pH 7.0) and 2.5 ml of 1% potassium ferricyanide. The mixtures were incubated at 50°C for 20 min. After that, 2.5 ml of 10% trichloroacetic acid and 2.5 ml of distilled water were added to the mixtures. Absorbance at 700 nm was measured (UVmini-1240, Shimadzu Corp., Japan). Reducing power was indicated by the changes in absorbance values. Higher absorbance values indicate stronger reducing power for the samples.

### Statistical Analysis

Statistical analyses were performed using SPSS (version 23, IBM Corporation, Armonk, New York, USA). Comparison analyses were performed using one-way ANOVA, and the least significant differences (LSDs) at 0.05 level were determined to indicate the significant differences. All the data were presented as the mean ± SD. Graphs were performed using SigmaPlot 12.5 (Systat Software Incorporation, San Jose, California, USA).

## Results

### Fruit Appearance and Browning Index During Storage

**Figure 1:**
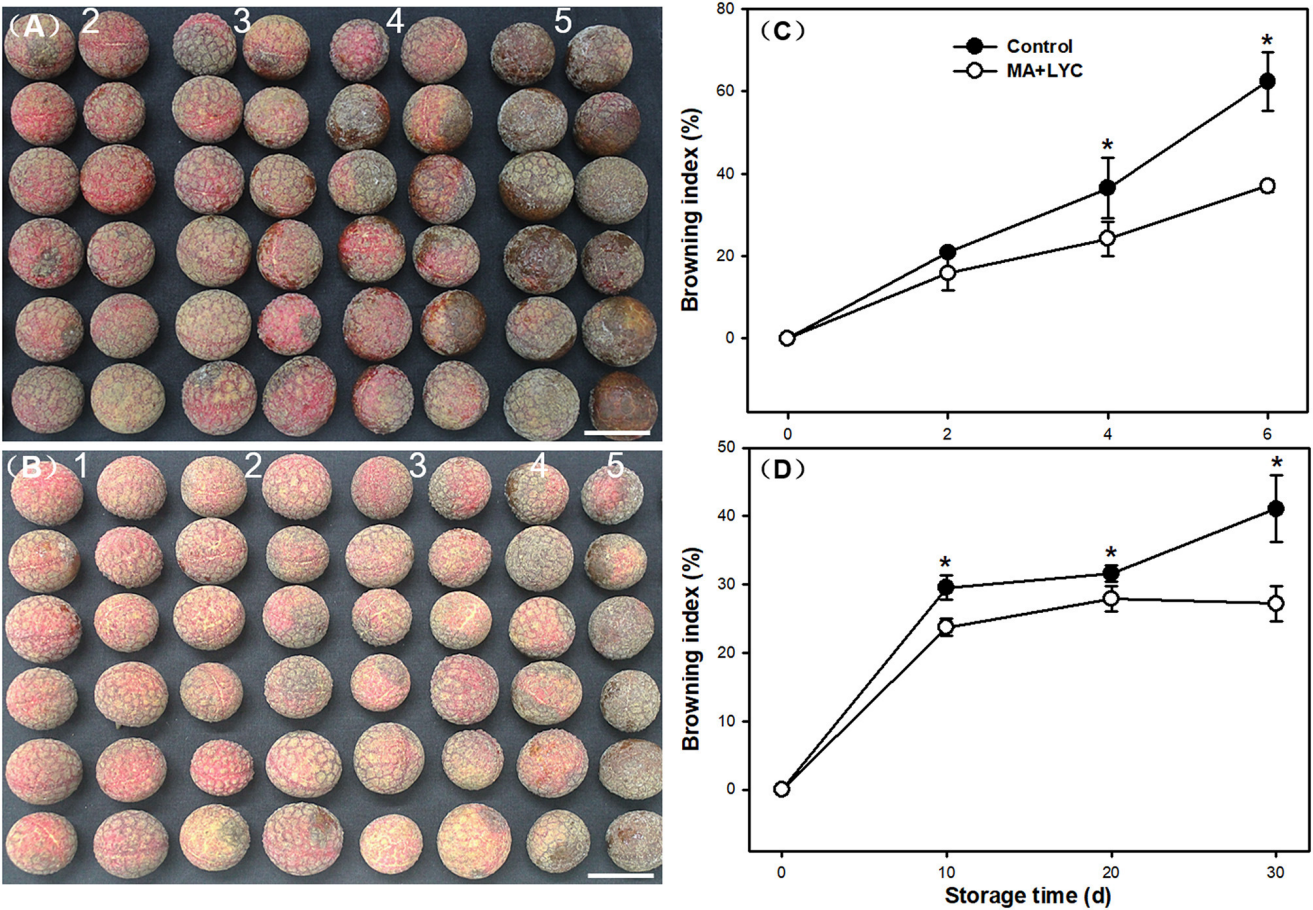
Appearance and the browning index changes in litchi fruit during storage. Appearance differences in litchi fruit taking samples at 6 d stored at ambient atmosphere for samples in **(A)** control, and **(B)** MA and LYC treatments. Changes in browning index in litchi fruit stored under **(C)** room and **(D)** low temperature during storage. Numbers in graph **(A)** and **(B)** indicate the browning scale. Scale bars in **(A)** and **(B)** indicate 2 cm. Asterisks in **(C)** and **(D)** indicate the significant difference of means at 0.05 level.

Pericarp browning developed following the senescence of fruit. As shown in Figure 1A, the visual appearance of litchi fruit browned severely after 6 days stored at RT (25°C). The combined treatments of MA and LYC exhibited potential effects in delaying the rapid browning process (Figure 1B). Browning index (BI) indicating the overall browning degree of fruit, increased gradually under RT during storage (Figure 1C). LT (4°C) storage slowed down the rapid browning process, which could be stored for up to 30 days (Figure 1D). In control, pericarp browning increased rapidly after 4 days at RT and 20 days at LT; the BI changed from 1.93 to 3.47 and from 1.53 to 2.05, respectively. However, the increase of BI was depressed by MA and LYC treatments, which raised from 1.31 to 2.65 at RT, and 1.33 to 1.36 at LT, respectively (Figures 1C,D). Generally, the treatments of MA and LYC exhibited efficiency in inhibiting the rapid browning development of pericarp browning under both RT and LT storage.

### MDA Content and Relative Leakage Rate Changes During Storage

**Figure 2:**
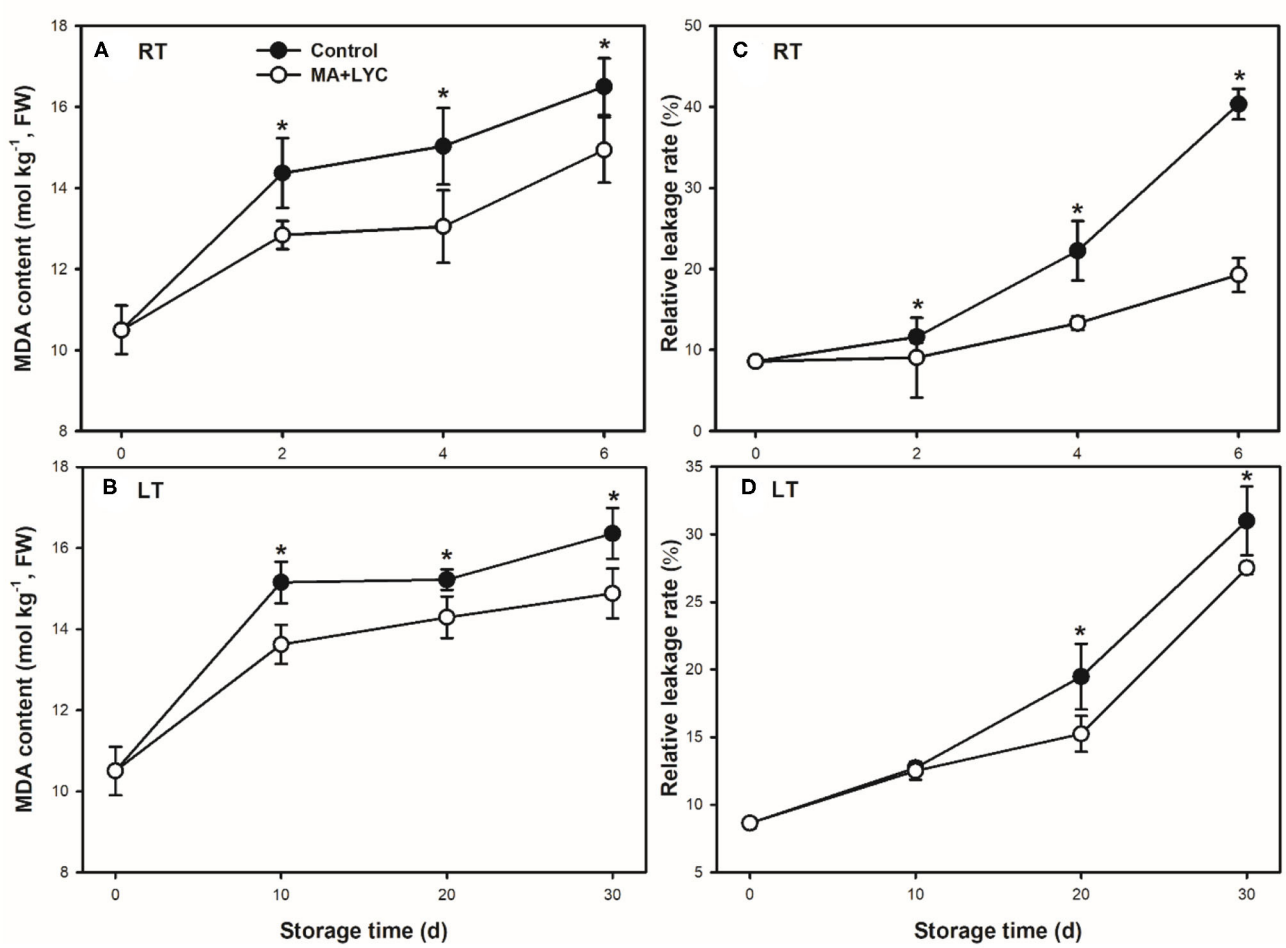
The membrane integrity indicated by the parameters of malondialdehyde (MDA) content and relative leakage rate stored at room temperature (RT) (25°C) and low temperature (LT) (4°C). MDA content changes at RT **(A)** and LT **(B)**, and relative leakage rate changes at RT **(C)** and LT **(D)**. Data are represented by mean values with the SD. Asterisk indicates the significant difference of means at 0.05 level.

The membrane integrity indicated by changes of MDA accumulation in cells and the relative leakage rate were determined. As shown in Figure 2, the cellular production of MDA and membrane permeability increased continuously following the extent of storage time under both RT and LT conditions. Overall, treatments of MA and LYC reduced the accumulation of MDA and increased of relative leakage rate (Figure 2). MDA production increased rapidly in the early 2 days at RT and 10 days at LT and then decreased gradually. The relative leakage rate increased slowly in the early several days, while increased rapidly thereafter. The relative leakage rate was 2- and 1.2-fold higher in control, as compared to that in treated fruits (Figures 2C,D).

### Content of Main Antioxidants in Fruit Pericarp

**Figure 3:**
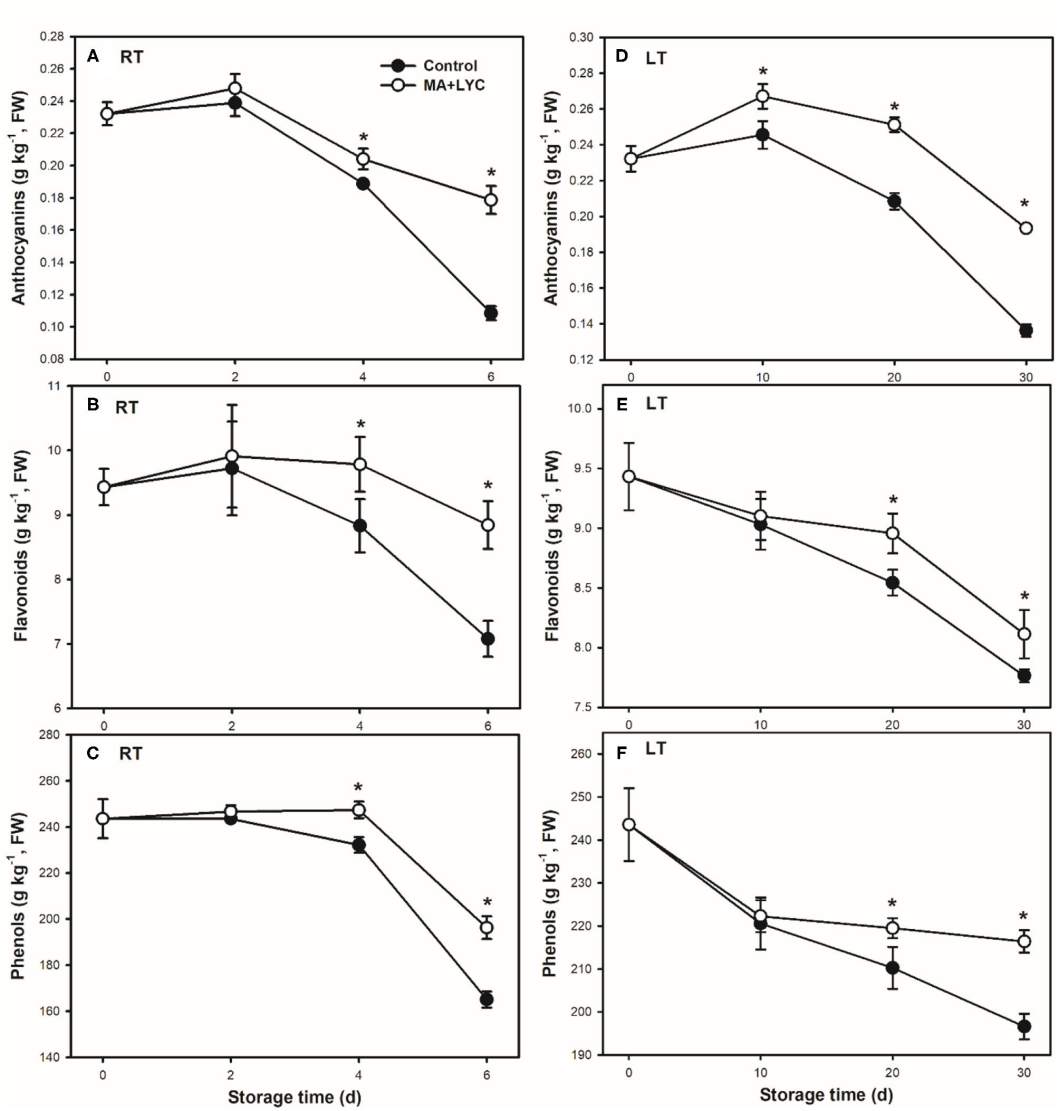
The content of polyphenols changes following the ripening and senescence of litchi fruit during storage. **(A,B)** The content of anthocyanins; **(C,D)** are the content of flavonoids; and **(E,F)** are the content of total phenols stored at RT (25°C) and LT (4°C), respectively. Data are represented by mean values with the SD. Asterisk indicates the significant difference of means at 0.05 level.

In litchi fruit, the abundant polyphenols, namely, the anthocyanins and flavonoids accumulated as the main antioxidants. In general, the total content of phenols, flavonoids, and anthocyanins exhibited decreased trends during storage (Figure 3). The total content of phenols decreased slowly in the early 4 days at RT and 20 days at LT, while decreasing rapidly thereafter, which was consistent with the change tends of browning development. Similarly, the treatments of MA and LYC retarded the decline of these above antioxidants. As compared to control, treated fruit contained 1.8-, 1.3-, and 1.3-fold higher anthocyanins, flavonoids, and total phenols, respectively, at 6 days under RT (Figures 3A–C). Similar change trends with 1.5-, 1.2-, and 1.2-fold higher for the content of anthocyanins, flavonoids, and total phenols, respectively, were in treated fruit than that in control under LT storage (Figures 3D–F).

### Enzyme Activity of PPO and POD Changes During Storage

**Figure 4:**
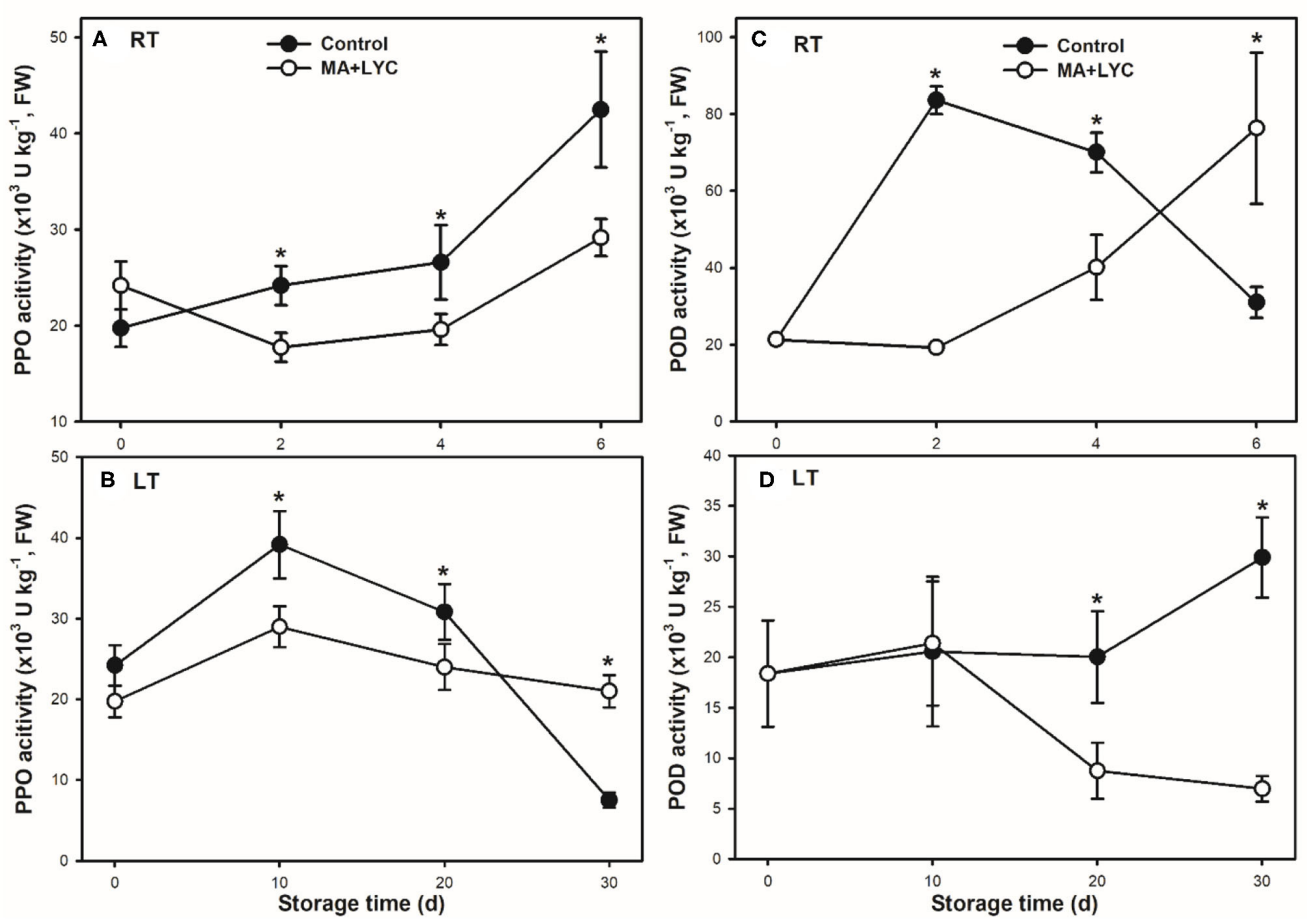
Enzyme activities of PPO **(A,B)** and POD **(C,D)** in litchi pericarp stored at RT (25°C) and LT (4°C), respectively. Data are represented by mean values with the SD. Asterisk indicates the significant difference of means at 0.05 level.

Enzyme activity of PPO and POD were analyzed to elucidate the enzymatic oxidation browning in litchi fruit during storage. Generally, the activity of PPO increased continuously under RT, while increased to peak at 10 days and declined thereafter under LT during storage (Figures 4A,B). Compared to control, the combined treatments of MA and LYC slowed down the fast changes of PPO activity. Under RT, the activity of PPO was lower in treated fruit than control through the whole storage time. In contrary to the stable levels in treated samples, PPO activity increased rapidly at 10 days, whereas declined sharply after 20 days in control under LT storage (Figures 4A,B).

The activity of POD peaked at 2 days and then declined gradually, which exhibited significantly higher in control than in treated fruit at 4 days under RT. PPO activity in treated fruit increased through the whole storage period (Figure 4C). POD activity maintained stable levels until 20 days and increased thereafter in control while decreasing after 10 days in treated fruit under LT storage (Figure 4D). The combined treatments of MA and LYC depressed the fast changes of enzyme activity of PPO and POD under both RT and LT conditions.

### Scavenging Activity of DPPH Radicals and Reducing Power

**Figure 5:**
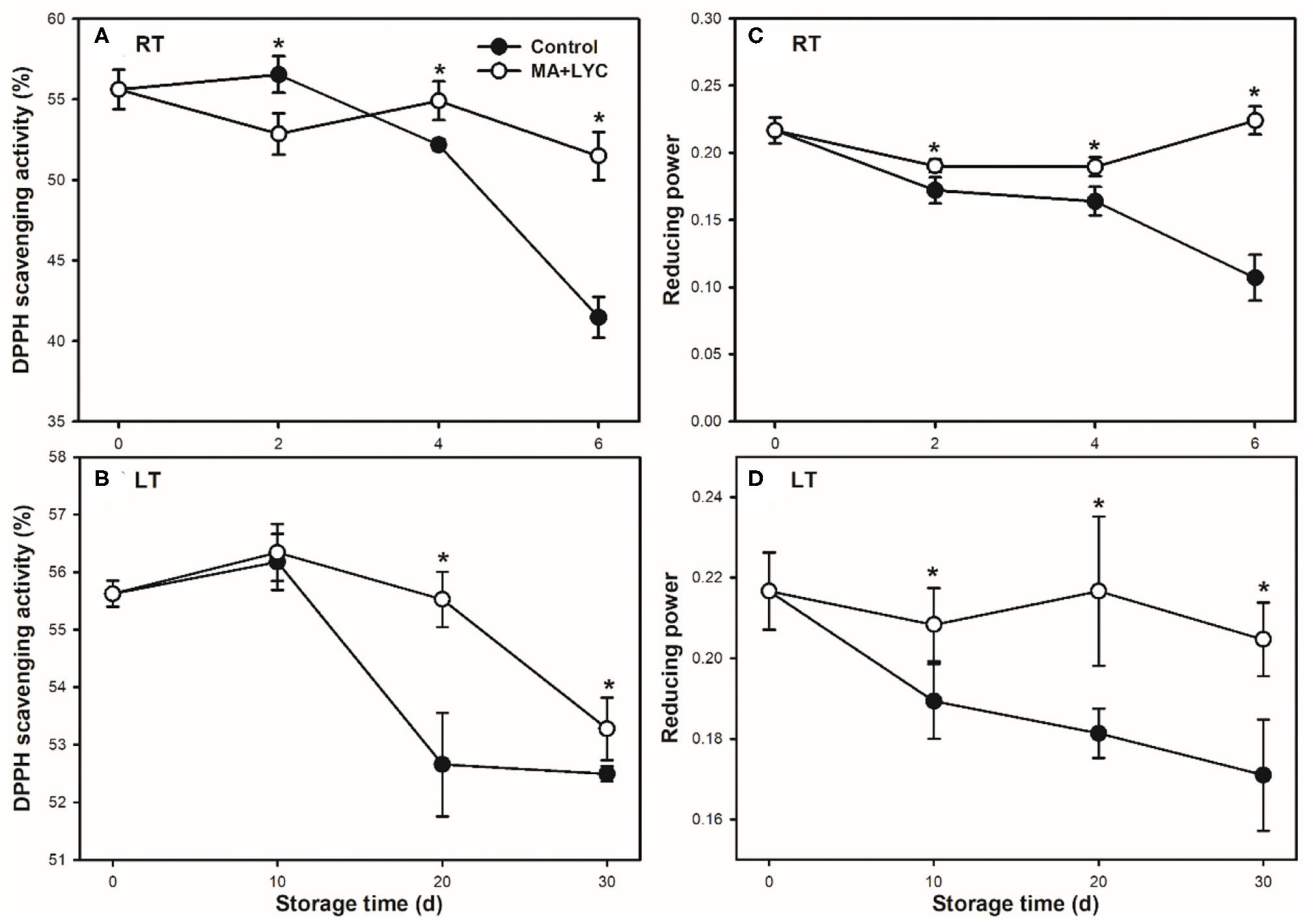
Non-enzymatic antioxidant activities of DPPH radical scavenging capacity **(A,B)** and reducing power **(C,D)** in litchi stored at RT (25°C) and LT (4°C), respectively. Data are represented by mean values with the SD. Asterisk indicates the significant difference of means at 0.05 level.

The non-enzymatic antioxidant activity was indicated by the scavenging activity of DPPH radicals and reducing power. Following the senescence of fruit after harvest, decreased trends for the scavenging activities of DPPH radicals and reducing power were detected at both RT and LT conditions during storage (Figure 5). MA and LYC treatments prevented the decreasing trend and maintained higher levels of DPPH radical scavenging activity and reducing power than that in control during storage (Figure 5). These results suggested that the pericarp tissues possessed a stronger electron-donating capability and scavenging activity of hydroxyl radicals in treated fruit.

## Discussion

Pericarp browning is one of the most serious problems that impact the marketable value for litchi fruit after harvest. The browning of pericarp has been widely reported as the result of the oxidation of polyphenols with or without enzyme catalysis (25). Litchi fruit is susceptible to environmental factors, which exhibited rapid browning at room ambient (26). Though the storage time can be extended under LT, dark-brown develops with the extent of storage, especially after being removed from cold ambient (21). Therefore, it is meaningful to search methods and technologies to alleviate the browning in litchi fruit after harvest. In this study, application of MA and LYC, the organic acid combined with antioxidant treatments exhibited potential effects in delaying the development of browning in litchi under both RT and LT storage. It is consistent with the previous reports that the organic acids such as citric acid, oxalic acid (4, 11), and antioxidants of apple polyphenol (9) are efficiently able to inhibit pericarp browning in litchi.

**Figure 6:**
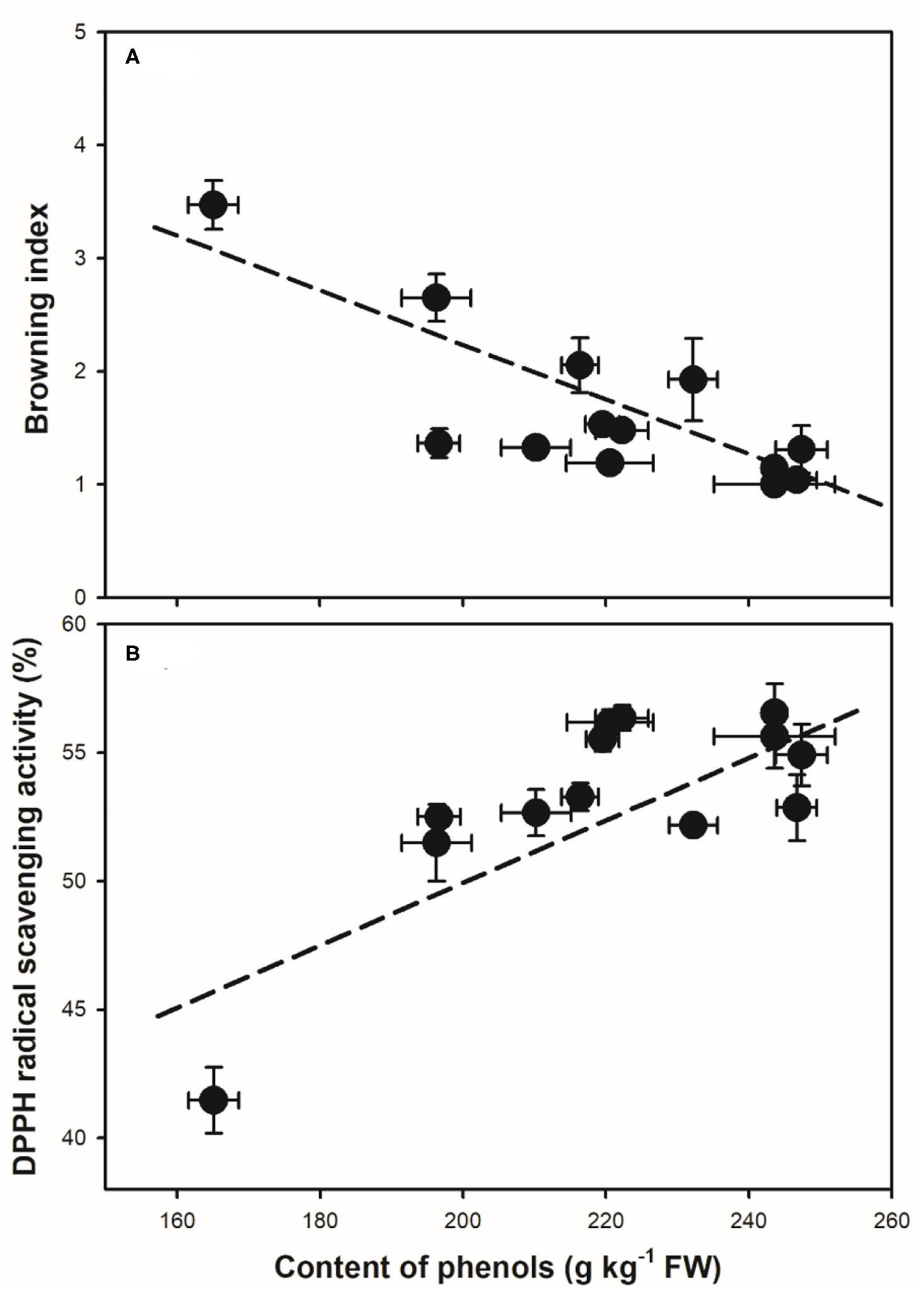
Correlation analysis between the content of pericarp phenols and the changes in **(A)** browning index (*r* = −0.83, *p* < 0.01), and **(B)** the DPPH radical scavenging capacity (*r* = 0.78, *p* < 0.01) during storage. Data of mean values with the SD were used in the correlation analysis.

The application of organic acids was also reported to affect the changes in pH and activity of oxidation-related enzymes and the antioxidant activity in fruit during storage. The changes in pericarp pH were reported as an indicator for the browning process in litchi (27). The abundant anthocyanins and activity of oxidation-related enzymes of PPO and POD were indicated to be affected by the changes in pericarp pH in litchi (4). As a result, MA treatment might be on one hand to alter the microenvironment pH in the pericarp, which further affected the pericarp polyphenol content and oxidation-related enzymes (Figures 3, 4). The capacity in inhibiting the accumulation of reactive oxygen species (ROS, O_2−_, and H_2_O_2_) could also be maintained by organic acid treatments in fruits after harvest (11, 16, 24). This will help to enhance the antioxidant activity. Meanwhile, as one of the main nutritional compounds in many crops varying in different species, cultivars, and organs, LYC is regarded as an important biological constituent and antioxidant (28). In addition to MA, the combined treatment of LYC might provide potential effects *via* its antioxidant capacity, which has been explored to extend the oil storage (19) and to induce the tolerance of chilling injury in grapefruit (29). Accordingly, similar to previous reports, MA might also take part in regulating the antioxidant capacity, thus, together with LYC to alleviate the oxidation browning process in litchi fruit under both RT and LT storage (Figure 6).

The polyphenols, namely, anthocyanins and flavonoids, and other phenolic components are the main constituents that influence the antioxidant activity in litchi (3, 30). In the treated litchi fruits, the decline of anthocyanins, flavonoids, and total phenols was markedly inhibited (Figure 3); and the DPPH radical scavenging capacity and reducing power were maintained (Figure 5), when compared with that in control. It has been reported that the development of the browning process in litchi was largely related to the content of antioxidants after harvest (3). Similarly, taking the results of control and the combined treatments of MA and LYC together, the content of phenols was negatively affected the changes in the pericarp browning index (Figure 6A); while was positively influencing the radical scavenging capacity (Figure 6B). It is highly comparable to those of previous reports by treatments or cultivar differences (3, 9), in which higher phenol content induced higher antioxidant activity, thus, alleviating the development of pericarp browning in litchi.

The oxidation of polyphenols occurred mainly in enzyme-activated changes and non-enzymatic modifications. During maturation and senescence of litchi fruit, the membrane integrity is susceptible to the external environment, which is indicated by relative leakage and lipid peroxidation products of MDA (21). The loss of membrane integrity induced by the senescence of fruit may further disturb the distribution of subcellular compartments and the oxidation-related enzymes (31). The destruction of the cell membrane in litchi pericarp may not only provoke the degradation of non-enzymatic phenols, i.e., anthocyanin substances but also stimulate the compartmentalization of oxidases such as PPO and POD. Once these enzymes contact the main substances of phenols, the enzymatic oxidation occurred rapidly (9, 25). The oxidation substances are further transformed into a black quinolone-polymer, and pericarp developed in completely dark brown (25). Though LT depresses the changes in the activity of these oxidation enzymes, the distribution of enzymes and substrates is susceptible to be disturbed with the senescence in litchi fruit. LT storage could obviously delay the shelf-life, while chilling injury symptoms occurred once the fruit is removed to RT ambient (21). In this study, the treated fruits exhibited a slower MDA accumulation rate and changes in relative leakage rate, as compared to control under both RT and LT (Figure 2). The combined treatments exhibited potential effects in maintaining the membrane integrity in litchi during storage. This could further prevent the interactions between oxidation enzymes and the phenols to slow down the activity changes in PPO and POD (Figure 4), thus, inducing slower pericarp browning in litchi fruit during storage.

**Figure 7:**
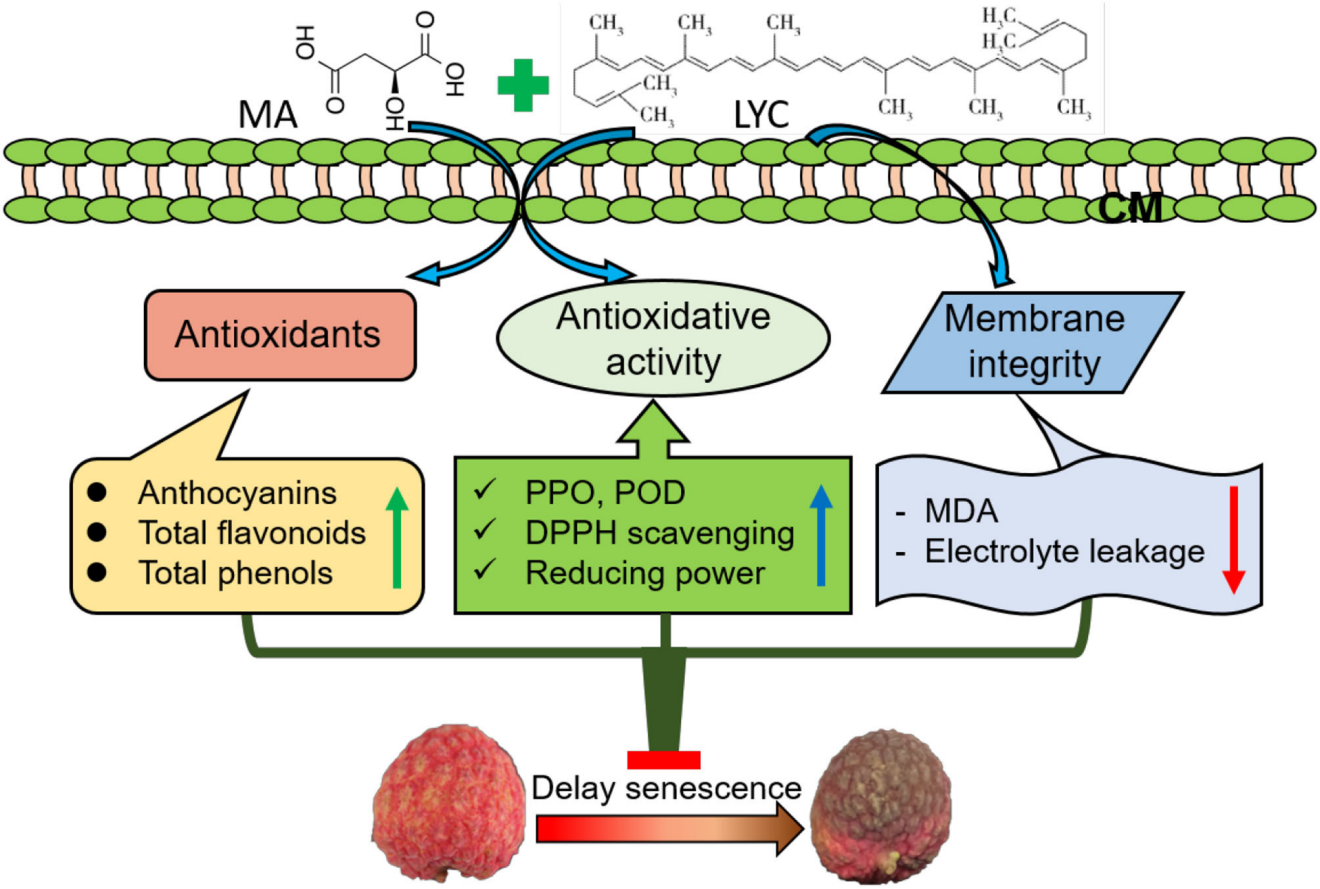
The potential preliminary mechanism for the regulation of combined treatment of MA an LYC on the pericarp browning in litchi fruit by maintaining the antioxidants level and antioxidant activity and the membrane integrity during storage.

In conclusion, the application of MA combined with LYC exhibited efficiency in alleviating the development of browning in litchi fruit under both RT and LT storage. Similar to the reported treatment of organic acids, i.e., oxalic acid, citric acid, and ascorbic acid, MA treatment together with LYC maintained membrane integrity and the contents of antioxidants, namely, total phenols, anthocyanins, and flavonoids. This further prevented the decline of antioxidant activity such as retarding the increase of PPO and POD to reduce the oxidation of phenols and inhibiting the decrease of the non-enzymatic oxidation such as radical scavenging activity and reducing power. Accordingly, treatment of MA and LYC provided a potential application way using natural compounds from plants to inhibit the browning and senescence of litchi fruit after harvest (Figure 7). Further work on the potential application of these treatments combined packages to optimize the postharvest quality maintenance in litchi is necessary to be explored.

## Data Availability Statement

The original contributions presented in the study are included in the article/supplementary material, further inquiries can be directed to the corresponding author.

## Author Contributions

HH designed the project, performed most of the experiments, analyzed data, and prepared the manuscript. LW and FB took part in part of the experiment and data analysis. XX provided fruit materials and helped to improve the manuscript. All authors have approved the final version of the manuscript.

## Funding

This work was supported by the Joint Funds of Basic and Applied Basic Research Foundation of Guangdong Province (2019A1515110611); Common Technical Innovation Team of Guangdong Province on Preservation and Logistics of Agricultural Products (2021KJ145); Funding of Rural Revitalization Special Strategic for Agricultural Improvement in Guangdong Province (TS-1-1); Funding of young scientist cultivation for Institute of Fruit Tree Research, Guangdong Academy of Agricultural Sciences (2021-107); Funding from State Key Laboratory for Conservation and Utilization of Subtropical Agro-bioresources (FKLCUSA-b201916); the Foundation of Guangxi Key Laboratory of Fruits and Vegetables Storage-Processing Technology (2021-01); Funding for Excellent Young Scientists of Guangdong Academy of Agricultural Sciences (R2021YJ-YB1005); special fund for scientific innovation strategy-construction of high level Academy of Agriculture science of Guangdong Academy of Agricultural Sciences (R2019QD-012), and Research and Development Program in Key Fields of Guangdong Province (2020B0202080003).

## Conflict of Interest

The authors declare that the research was conducted in the absence of any commercial or financial relationships that could be construed as a potential conflict of interest.

## Publisher's Note

All claims expressed in this article are solely those of the authors and do not necessarily represent those of their affiliated organizations, or those of the publisher, the editors and the reviewers. Any product that may be evaluated in this article, or claim that may be made by its manufacturer, is not guaranteed or endorsed by the publisher.
